# Investigation of Hydrothermally Stressed Silicone Rubber/Silica Micro and Nanocomposite for the Coating High Voltage Insulation Applications

**DOI:** 10.3390/ma14133567

**Published:** 2021-06-25

**Authors:** Faiza, Abraiz Khattak, Safi Ullah Butt, Kashif Imran, Abasin Ulasyar, Asghar Ali, Zuhair Subhani Khan, Azhar Mahmood, Nasim Ullah, Ahmad Aziz Alahmadi, Adam Khan

**Affiliations:** 1 School of Natural Sciences, National University of Sciences and Technology (NUST), Sector H-12, Islamabad 44000, Pakistan; faizaijaz710@gmail.com (F.); dr.azhar@sns.nust.edu.pk (A.M.); 2 U.S.-Pakistan Center for Advanced Studies in Energy, Department of Electrical Power Engineering, National University of Sciences and Technology (NUST), Sector H-12, Islamabad 44000, Pakistan; safibutt541@gmail.com (S.U.B.); kashifimran@uspcase.nust.edu.pk (K.I.); 3 U.S.-Pakistan Center for Advanced Studies in Energy, Department of Energy Systems Engineering, National University of Sciences and Technology (NUST), Sector H-12, Islamabad 44000, Pakistan; engr.abasin@gmail.com (A.U.); asghar@uspcase.nust.edu.pk (A.A.); zskhan@ces.nust.edu.pk (Z.S.K.); 4 Department of Electrical Engineering, College of Engineering, Taif University KSA, P.O. Box 11099, Taif 21944, Saudi Arabia; nasimullah@tu.edu.sa (N.U.); aziz@tu.edu.sa (A.A.A.); 5 Department of Electronics Engineering, University of Engineering and Technology (UET) Peshawar (Abbottabad Campus), Abbottabad 22010, Pakistan; adamkhan@uetpeshawar.edu.pk

**Keywords:** high voltage, modern insulation, aging, polymer, nanocomposites

## Abstract

Silicone rubber is a promising insulating material that has been performing well for different insulating and dielectric applications. However, in outdoor applications, environmental stresses cause structural and surface degradations that diminish its insulating properties. This effect of degradation can be reduced with the addition of a suitable filler to the polymer chains. For the investigation of structural changes and hydrophobicity four different systems were fabricated, including neat silicone rubber, a micro composite (with 15% micro-silica filler), and nanocomposites (with 2.5% and 5% nanosilica filler) by subjecting them to various hydrothermal conditions. In general, remarkable results were obtained by the addition of fillers. However, nanocomposites showed the best resistance against the applied stresses. In comparison to neat silicone rubber, the stability of the structure and hydrophobic behavior was better for micro-silica, which was further enhanced in the case of nanocomposites. The inclusion of 5% nanosilica showed the best results before and after applying aging conditions.

## 1. Introduction

The development of polymeric insulators intrigued the interest of researchers in this field due to the achievement of superior properties, such as light weight, high flashover voltages, resistance to contaminations, and high dielectric strength, as compared to ceramic insulators [1,2]. Polymers such as Ethylene Propylene Diene Monomer (EPDM), Polyamide, Silicone Rubber (SiR) and Ethylene-Vinyl Acetate (EVA) are being used for electrical, construction, dielectric, and outdoor/indoor high voltage applications due to their beneficial mechanical, electrical, thermal, and dielectric properties. Among the abovementioned polymers, silicone rubber (SiR) is an important insulating polymer because it has a unique aging behavior among the polymeric insulators. It shows both degradation and recovery during environmental aging [3,4]. Hard silicone rubber or high temperature HTV has high molecular weight due to comparatively long polymeric chains and finds its application in high voltage composite insulators as a housing material [5,6,7]. The excellent weathering and heat resistance characteristics of SiR make it suitable for outdoor insulation. It is also coated on ceramic insulators to improve their hydrophobicity and minimize the leakage current [8,9]. Despite these characteristics, SiR suffers from degradation in outdoor environments that needs to be addressed. The main drawback of polymeric insulators is their organic nature, which causes degradation of the structure under different environmental stresses such as UV light, fog, humidity, temperature, and acid rain [10,11,12,13]. The synergistic effect of these environmental stresses contributes to the deterioration of the physiochemical and thermomechanical properties of polymeric insulators. This degradation in performance hinders the outdoor usage of these insulators and highlights the importance of materials that can maintain adequate performance despite environmental stresses. An efficient way to ameliorate the performance of polymeric insulators, as reported by many authors, is the formation of composites [14,15]. The composites are formed by the addition of inorganic oxides in the polymer.

Polymeric composites replace polymeric insulators because they offer substantial enhancement in electrical, mechanical and thermal properties [16,17]. Preparation of composites must be carried out with extreme care because their performance depends on the level of preparation efficiency. There are numerous inorganic fillers whose addition offers better performance than neat polymers, e.g., alumina, titania and silica. Alumina and titania enhance anti-erosion, anti-tracking and thermal conductivity, whereas silica improves mechanical and dielectric properties along with erosion and tracking. Additionally, silica is the cheapest and easily available. Furthermore, silica is an appropriate inorganic filler to enhance the properties of polymeric insulators [18,19,20].

Several significant studies have been carried out on the aging investigations of silicone rubber under various environmental stresses. The synergistic effect of UV light, salt, fog, humidity, acid rain and heat on the performance of silicone rubber and its composites was reported by Khattak et al. [21]. The hydrophobicity recovery phenomenon of both RTV and HTV silicone rubber under different stresses was investigated in various studies, implying that the transfer of Low Molecular Weight (LMW) components from material bulk to the surface is the main reason for the recovery of hydrophobicity of silicone rubber [22,23,24,25]. Ahmadi-veshki et al. concluded that pollution and humidity increased the probability of occurrence of flashover [26]. Wang et al. described that the hydrophobicity of SiR Insulator became worse and samples became rigid after UV treatment [10]. Yuan and co-workers studied the dielectric loss of SiR/SiO_2_ composites under AC corona at different AC voltages and observed that change in dielectric loss remained under 1% after 24 days of corona aging at 3 kV [27]. Degradation of silica filled silicone rubber under accelerated ultraviolet weathering conditions has also been reported [28]. The dielectric properties of newly manufactured and 500 kV composite insulators operated shed and housing material was determined under hydrothermal conditions (20 °C, 90% humidity). The moisture content saturated after increasing initially for both materials and dielectric loss followed the same trend [29]. A large number of studies investigated the behavior of HTV SiR, whereas very little literature is available for RTV SiR, which is widely recommended for coatings of high voltage insulators.

This study focuses on assessing the change in hydrophobic behavior of RTV SiR based nano and micro composites under accelerated hydrothermal aging conditions. Moreover, detailed structural analysis was performed by using Fourier Transform Infrared (FTIR) spectroscopy and Scanning Electron Microscopy (SEM) analyses.

## 2. Material Procurement and Sample Preparation

### 2.1. Material Procurement

The Room Temperature Vulcanized (RTV-615) silicone rubber, nanosilica (12 nm), and micro-silica (5 µm) used in this work were acquired from Lanxess AG Chemicals, Leverkusen Germany, Degussa, New Jersey, USA and Wuhan Newreach Chemicals in Wuhan, China, respectively.

### 2.2. Sample Preparation

Silicone rubber/silica composites were prepared with percentage weight (wt%) formulation of polymer and fillers, i.e., SNC 2.5 are the samples having 2.5 gm of nanosilica and 97.5 gm of RTV 615 silicone rubber (with 86.36 gm of RTV 615-A (base polymer), and 8.64 gm of RTV 615-B (curator)). A ratio of 10:1 was maintained between the base polymer and curator in the samples. The sample preparation was carried using a shear mixer along with a sonicator. The composition of the prepared samples was 2.5 wt% of nanosilica, 5 wt% of nanosilica and 15 wt% of microsilica. A summary of the formulated samples is presented in Table 1. Fumed silica filler was treated with ethanol to increase its dispersion in the polymer matrix [30]. The temperature of silica fillers was maintained at 160 °C for 16 h before preparation and SiR was placed in a Memmert vacuum oven at 460 mm-Hg for few hours. Initially, the mixing of the dry fillers RTV 615-A was performed at a low speed of 3000 rpm so that the fillers receive proper wetting, and then mixing was carried out at a maximum speed of 5000 rpm until all visible lumps in the mixture disappeared. Low speed mixing of RTV 615-B was then carried out by a sonicator at low speed to achieve maximum uniformity. In the final step, the mixture was housed in a vacuum oven for degassing and debubbling at 27 mm-Hg. The prepared slurry was poured into the molds and retained at room temperature for 24 h. The samples were further cured at 90 °C in an oven for 4 h.

### 2.3. Aging Setup

**Figure 1:**
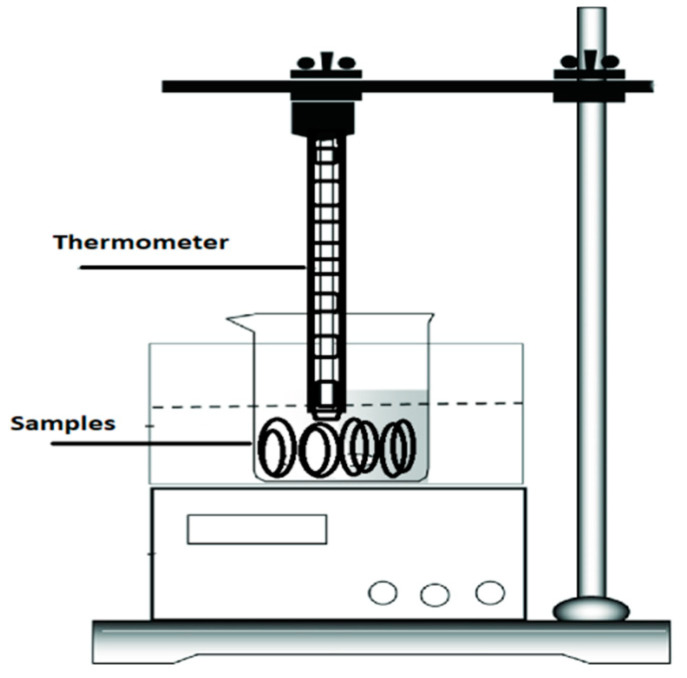
Hydrothermal aging setup.

The hydrothermal aging experiment was performed in a glass beaker. The beaker was placed on a hot plate and insulator samples were immersed in water. The temperature of the water was retained between 60 and 70 °C. The aging period of 1000 h was completed in five cycles; each cycle was 200 h long. The experimental setup is illustrated in Figure 1.

### 2.4. Analysis Techniques

#### 2.4.1. Hydrophobicity Classification

To probe the hydrophobic behavior of the prepared samples, Swedish Transmission Research Institute (STRI) classification was employed. The samples were sprinkled with tap water for 20 s and hydrophobicity was evaluated within 10 s by comparison of high-resolution images of samples against the standard STRI classification guide.

#### 2.4.2. Scanning Electron Microscopy (SEM)

Field Emission Scanning Electron Microscopy (FESEM) model MIRA3 TESCAN Zeiss Supra 55 VP, Kohoutovice, Czech Republic was used for morphological analysis. Since samples were non-conducting, carbon coating was used and samples were placed on the stubs for the analysis.

#### 2.4.3. Fourier Transform Infrared Spectroscopy (FTIR)

Bruker platinum ATR model Alpha, Bremen, Germany with a spectral range of 4000–500 cm^−1^ was used to perform the FTIR analysis. Analysis was carried out by placing samples on a diamond scanner, and after that their absorbance was measured against the wave number (cm^−1^).

## 3. Results and Discussion

### 3.1. Hydrophobicity Classification

**Figure 2:**
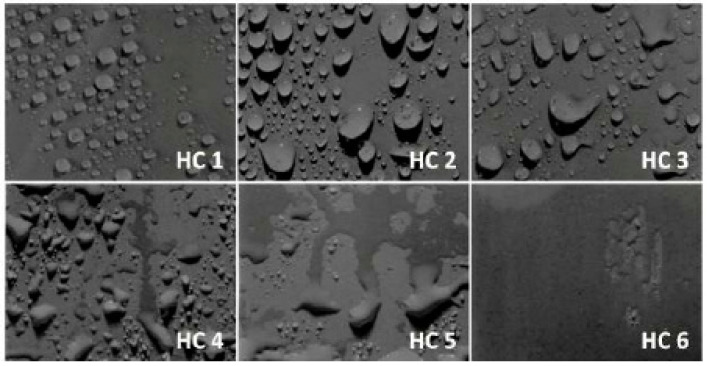
Hydrophobicity class Swedish transmission research institute (STRI) guide.

**Figure 3:**
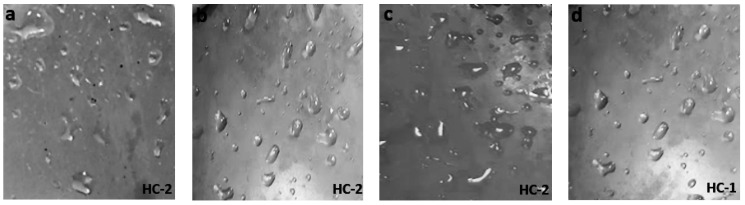
Images of un-aged (**a**) nSiR (**b**) SMC 15 (**c**) SNC 2.5 and (**d**) SNC 5.

**Figure 4:**
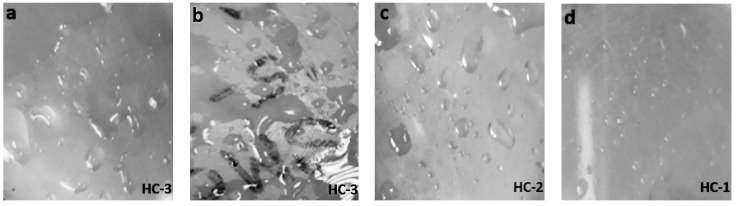
Images of aged (200 h) (**a**) nSiR (**b**) SMC 15 (**c**) SNC 2.5 and (**d**) SNC 5.

**Figure 5:**
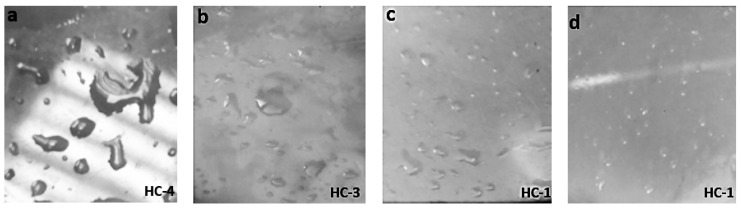
Images of aged (400 h) (**a**) nSiR (**b**) SMC 15 (**c**) SNC 2.5 and (**d**) SNC 5.

**Figure 6:**
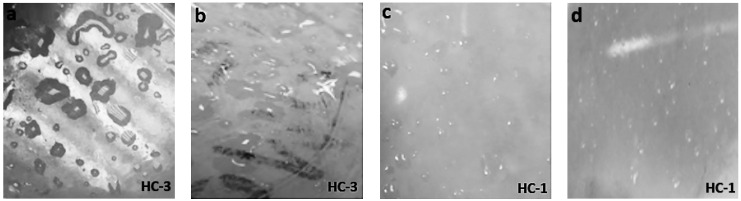
Images of aged (600 h) (**a**) nSiR (**b**) SMC 15 (**c**) SNC 2.5 and (**d**) SNC 5.

**Figure 7:**
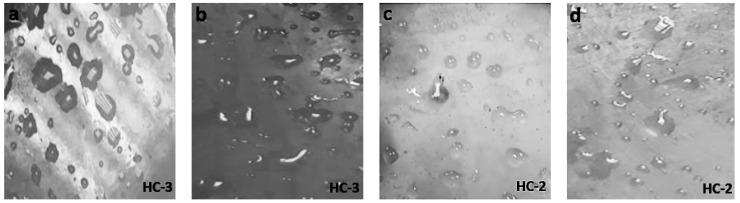
Images of aged (800 h) (**a**) nSiR (**b**) SMC 15 (**c**) SNC 2.5 and (**d**) SNC 5.

**Figure 8:**
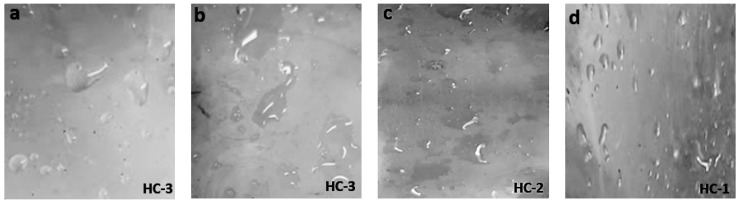
Images of aged (1000 h) (**a**) nSiR (**b**) SMC 15 (**c**) SNC 2.5 and (**d**) SNC 5.

**Figure 9:**
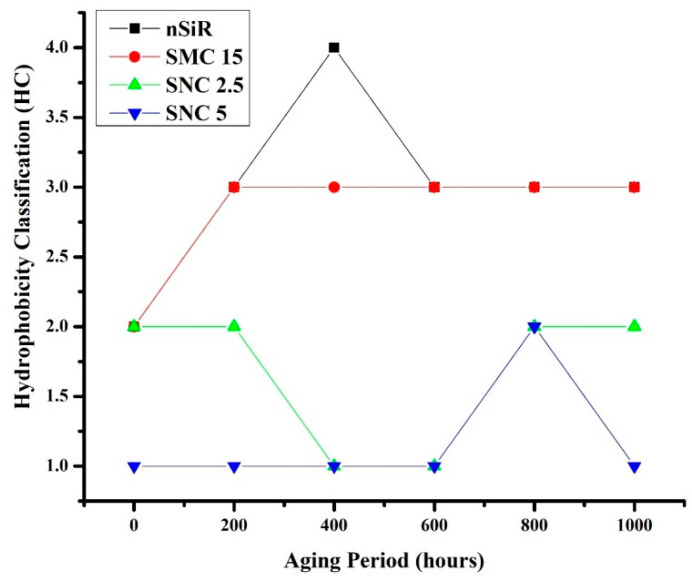
Variation in hydrophobicity with aging time.

The hydrophobic trends of all the samples were analyzed after each cycle and it was seen that they varied in an unpredictable manner with changes in concentration and size of the fillers. Swedish Transmission Research Institute (STRI) classification presents six hydrophobicity classes from HC-1 to HC-6, where HC-1 refers to the most hydrophobic and HC-6 to the most hydrophilic behavior. Figure 2 represents the standard images of STRI classification that were used to determine the hydrophobicity class of each sample. It was observed that Neat SiR showed significant variation of hydrophobic character, while micro-silica loaded samples retained their hydrophobicity better than nSiR. Furthermore, a decrease in hydrophobicity with increases in aging time can be clearly observed in Figure 3, Figure 4, Figure 5, Figure 6, Figure 7 and Figure 8. In comparison, nano-filler loaded samples showed less variation and retained their hydrophobicity class even after 1000 h of aging. All of the samples exhibited their best hydrophobicity class before aging, as shown in Figure 9, and loss in hydrophobicity was observed as samples were subjected to the aging stresses. This loss of hydrophobicity is due to the loss of low molecular weight components on the surface, which occurs due to wetting. It was noted that droplets were initially circular in shape, whereas their shape was distorted after aging due to the non-uniform surface energy. Before application of stresses, SNC-5 exhibited the best hydrophobic behavior (HC-01) among all samples. Nevertheless, all other samples also had a good water repellency class of HC-2, as shown in Figure 9. Neat SiR started from HC-02 and exhibited HC-03 in several observations. Although HC-4 was observed after 400 h, the hydrophobicity of nSiR recovered during the next cycles to HC-3. This behavior of loss and recovery in hydrophobicity of SiR has been reported by many authors and the primary reason considered for this unique behavior is the rotation of the polymer backbone chain and transfer of low molecular weight components from material bulk to the surface of insulators [31,32].

SMC 15 also showed HC-2 before aging and dropped to HC-3 after the first cycle but remained stable at HC-3 until 1000 h of aging. More stability was shown by SMC 15 as compared to neat silicone rubber. SNC 5 and SNC 2.5 showed greater stability than the micro filled sample and neat silicone rubber. SNC 2.5 showed HC-2 both before aging and at the end of the fifth cycle. However, temporary variations were observed at the end of a few intermediate cycles. In comparison, SNC 5 showed HC-1 before aging and retained it for 1000 h. Loss in hydrophobicity of SNC 5 was observed at 800 h, but it was recovered in the next cycle. SNC 5 showed excellent hydrophobicity (HC-1) that was retained even at the end of the aging period. The behavior of SNC 5 is attributed to the larger surface area provided by the inclusion of a nanofiller. The larger surface area provides better water repellency; the surface area increases with an increase in the concentration of nanofiller. Consequently, the hydrophobicity of samples with a greater concentration of nanofiller was higher. The variation trend of all samples can be seen in Figure 9.

### 3.2. Scanning Electron Microscopy (SEM)

**Figure 10:**
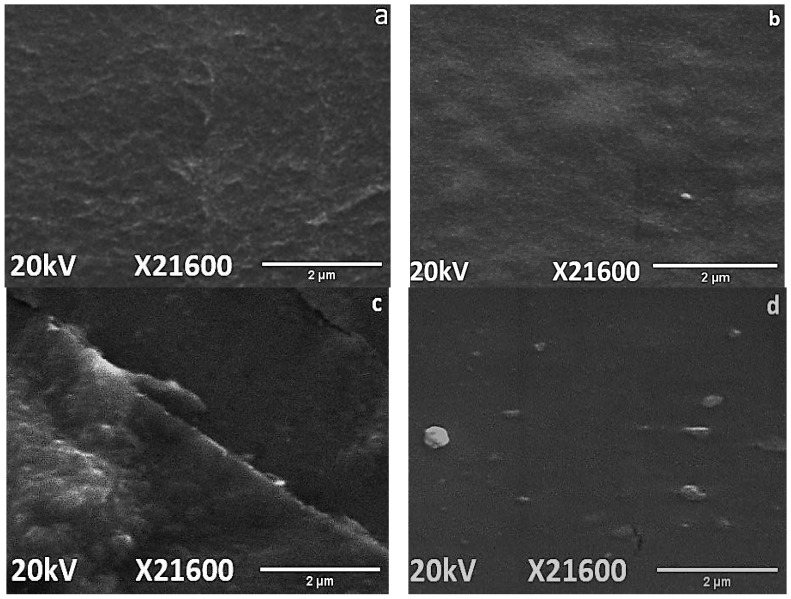
SEM images of virgin (**a**) nSiR (**b**) SMC 15 (**c**) SNC 2.5 and (**d**) SNC 5.

**Figure 11:**
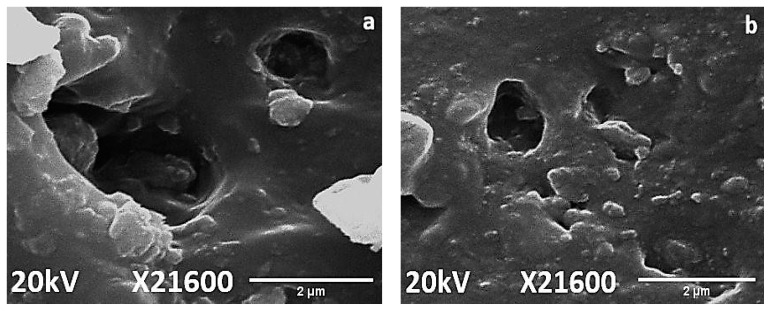

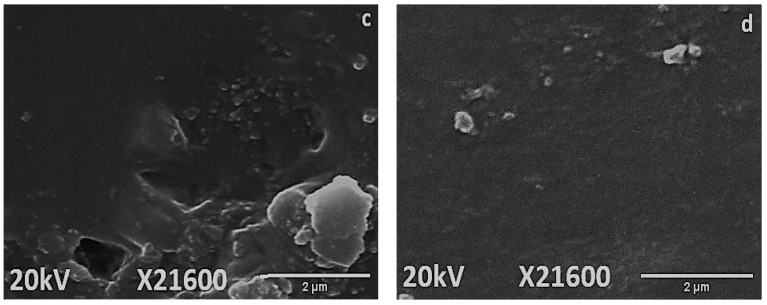
SEM images of 1000 h aged (**a**) nSiR (**b**) SMC 15 (**c**) SNC 2.5 and (**d**) SNC 5.

Surface topography and distortion due to aging was evaluated by SEM analysis for neat silicone rubber and its micro and nanocomposites. SEM images of virgin and 1000 h aged samples were captured from a distance of 2 µm. It was observed that all of the samples expressed smooth surfaces before aging, as shown in Figure 10. Moreover, Figure 11 shows surface degradation observed for all samples after applications of the aging conditions. However, the extent of degradation varied with the shape and concentration of the filler. Loss of material and holes after 1000 h of aging were lowest in the case of nanocomposites, as shown in Figure 11. In comparison to neat silicone rubber, SMC15 showed improved resistance to change in the structure against aging stresses. The better surface performance of SMC 15 may also be due to the abundance of the silanol group, which provides better intactness with the functional groups of SiR. Degradation at the surface in SMC was much lower than nSiR.

When a nano filler was used, degradation was further minimized because nanosilica provided a greater surface area to the functional groups of SiR [33,34]. However, SNC 2.5 expressed greater degradation and porosity than SNC 5, as seen in Figure 11. This is due to increased chain intactness in the case of SNC 5, which consequently reduced the distortion in surface morphology [35]. SEM images showed that a 5 wt%% addition of silica is optimum. It was concluded that SNC 5 showed the best resistance and least degradation of the surface against aging conditions as compared to the other samples. These results are in agreement with the hydrophobicity results.

### 3.3. Fourier Transform Infrared (FTIR) Spectroscopy

**Figure 12:**
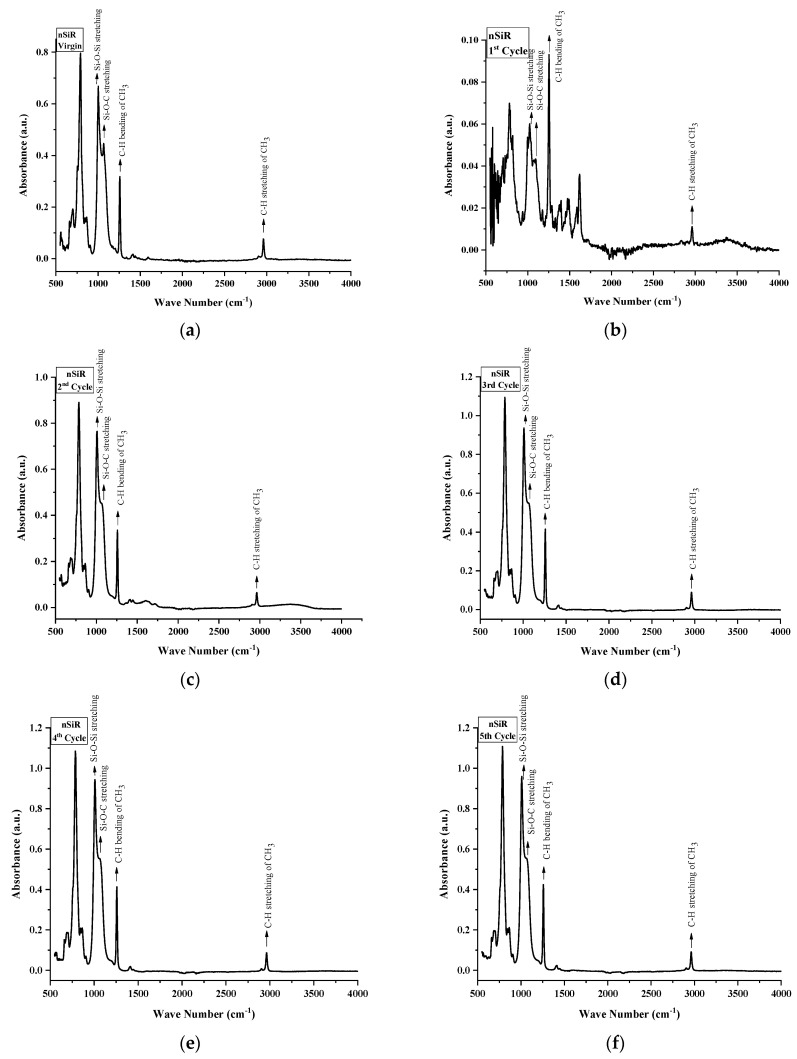
FTIR images of neat silicone rubber: (**a**) virgin; (**b**) after 200 h. (**c**) after 400 h; (**d**) after 600 h; (**e**) after 800 h; (**f**) after 1000 h.

**Figure 13:**
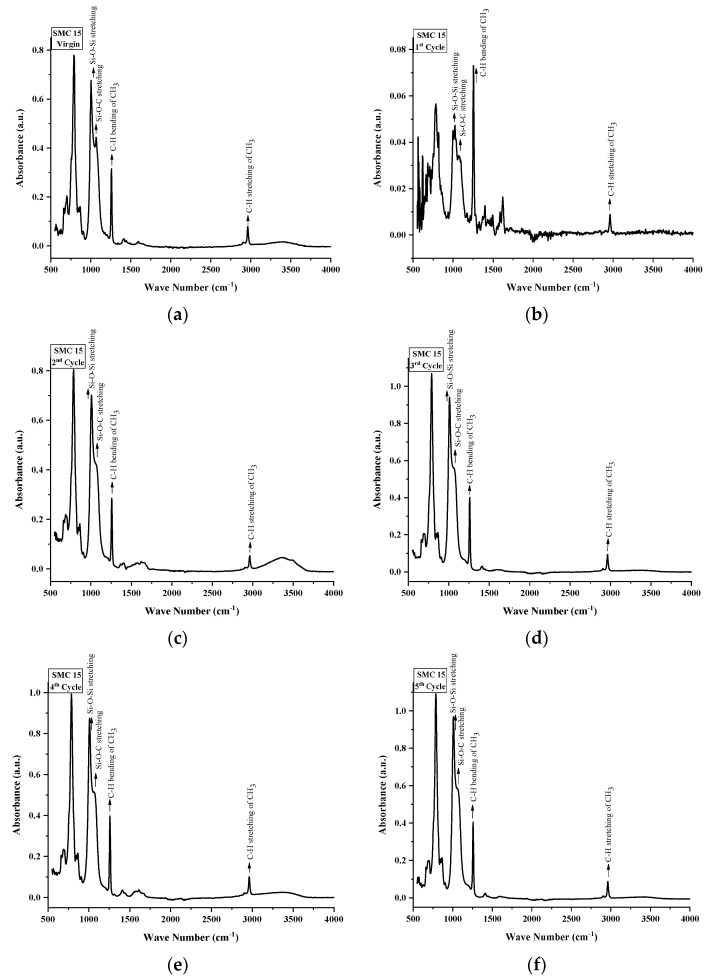
FTIR images of SMC 15: (**a**) virgin; (**b**) after 200 h. (**c**) after 400 h; (**d**) after 600 h; (**e**) after 800 h; (**f**) after 1000 h.

**Figure 14:**
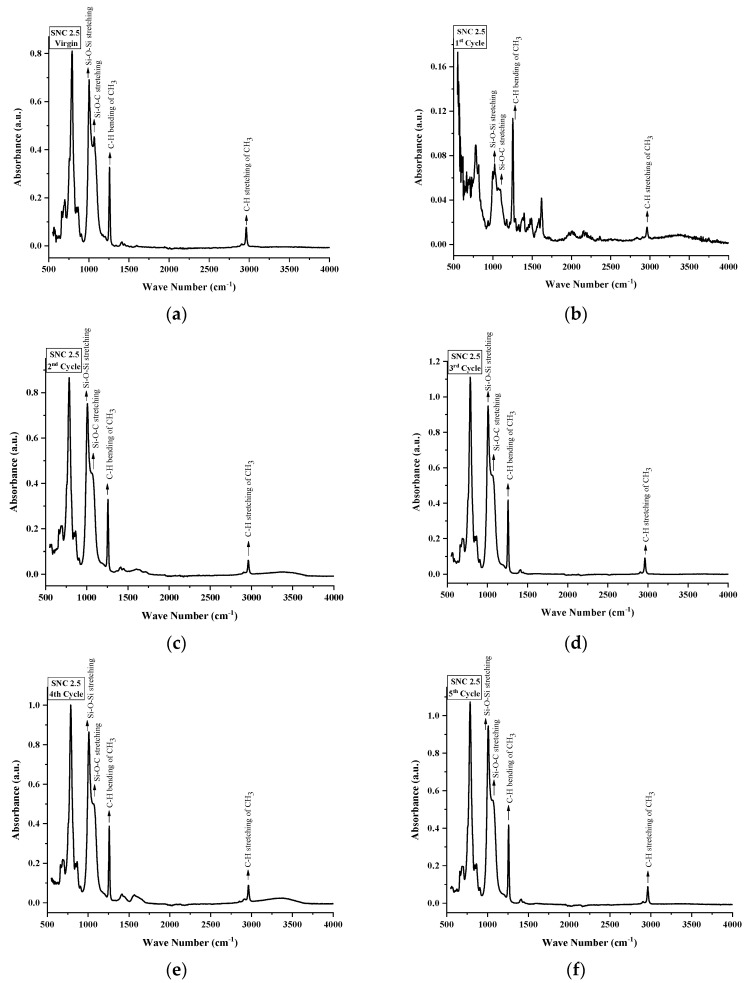
FTIR images of SNC 2.5: (**a**) virgin; (**b**) after 200 h. (**c**) after 400 h; (**d**) after 600 h; (**e**) after 800 h; (**f**) after 1000 h.

**Figure 15:**
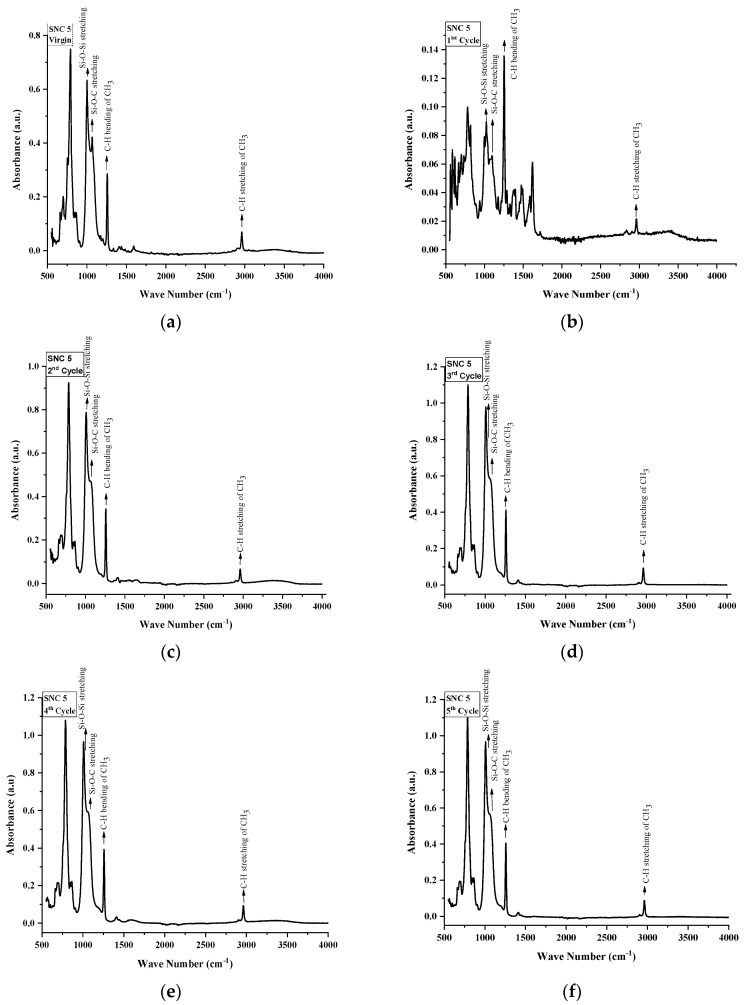
FTIR images of SNC: (**a**) virgin; (**b**) after 200 h. (**c**) after 400 h; (**d**) after 600 h; (**e**) after 800 h; (**f**) after 1000 h.

FTIR spectroscopy was used to inspect the structural changes in nSiR, SMC 15, SNC 2.5 and SNC 5. All of the samples were analyzed after each cycle in the absorption form. All samples exhibited noticeable structural changes. FTIR plots of nSiR, SMC 15, SNC-2.5 and SNC-5 are given in Figure 12, Figure 13, Figure 14 and Figure 15 respectively. The absorbance of prominent groups and their respective wave numbers are given in Table 2. As shown in shown in Table 3 In the case of neat silicone rubber, most important hydrocarbon groups like symmetric C–H stretching of CH_3_ at ~2963–2960 cm^−1^ showed an absorbance of 0.07 a.u., and a decrement of 85.71% and 14.28% occurred in the absorption peak of symmetric C-H stretching of CH_3_ after 100 and 200 h, respectively. However, it exhibited recovery in the fourth cycle and the final recorded absorbance of 0.07 a.u. was the same as that of the virgin sample as shown in Figure 12. All absorbance values for nSiR are given in Table 2.

After the first cycle, all samples went under excessive oxidation, as the peaks of C=C and C=O appeared at 1612 and 1725 cm^−1^, respectively. In addition, an substantial decrease in the absorbance of all of the bands was observed, but recovery was seen after the second cycle. The same trend was seen in the case of virgin SMC 15, as shown in Figure 13. The numerical values are also given in Table 4. However, some recovery was observed after the second cycle and an increase of 28.56% occurred after the third and fourth cycle. A final absorbance value of 0.08 was noted that was 14.28% greater than the one of virgin SMC 15. In the case of SNC 2.5 and SNC 5, however, degradations of 85.71% and 71.42%, respectively, in the peaks of CH_3_ stretching were seen after the first cycle, but recovery was observed after second cycle. Similarly, a 66.67% decrement in the peak of CH_3_ bending was seen for both SNC 2.5 and SNC 5 after the first cycle. However, both expressed recovery in the second cycle. It is clear from Figure 14 and Figure 15 that nanocomposites exhibited greater recovery after the second cycle than nSiR and micro composites, which implies that nanoparticles enhanced the stability of the composite. Numerical values for SNC-2.5 and SNC-5 are given in Table 5 and Table 6 respectively.

It is discernable that after the first cycle, all samples showed degradation, but the smallest deterioration was seen in the case of SNC 5. Si-CH_3_ symmetric bending (~1280–1255 cm^−1^) showed an absorbance of 0.3 a.u. for all of the samples, which showed degradation in the peak after the first cycle; however, after the second cycle recovery was observed, which was mostly maintained until the fifth cycle, and no decrement in this peak of samples was seen even at the end of the last cycle. However, after the first cycle, the decrement observed in the peaks of all samples was the smallest in the case of SNC 5.

**Figure 16:**
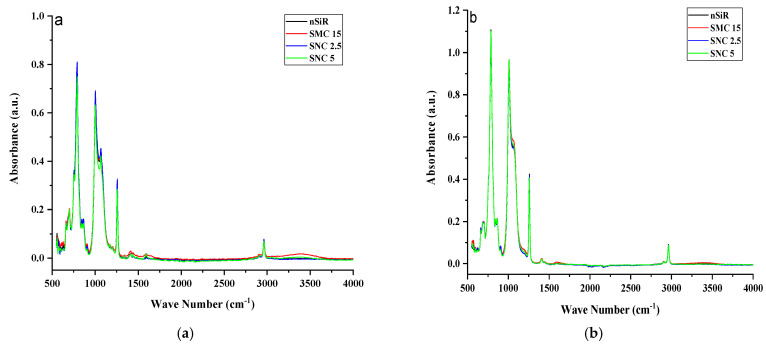
Overlay FTIR plots of samples (**a**) before aging and (**b**) after aging.

Overlay plots for better comparison are given in Figure 16.

Similarly, Si-O-C stretching (~1110–1050 cm^−1^) and Si-O-Si asymmetric stretching (~1130–1000 cm^−1^) showed a mostly similar trend for all the samples. Decrement and recovery in these peaks were found for all samples, but it is noteworthy that decrement was the smallest in the case of SNC 5. Therefore, it can be deduced from the above discussion that the inclusion of 5 wt% of nanosilica increased the stability of functional groups, as shown in Table 6.

## 4. Conclusions

For inspecting the structural stability and hydrophobicity under accelerated hydrothermal stresses, different composites of silicone rubber reinforced with silica (SiO_2_) micro (15%) and nano (2.5% and 5%) composites were prepared. The prepared samples were then subjected to an accelerated hydrothermal environment. FTIR, SEM, and hydrophobicity results were recorded after every 200 h and up to 1000 h. Variations in hydrophobicity and absorption peaks of prominent groups were observed during the aging period. Nanocomposites (SNC 2.5 and SNC 5) exhibited better performance as compared to nSiR and SMC 15. Among the nanocomposites, SNC 5 showed better hydrophobicity during the entire aging period. SEM results indicated that all SiR formulations suffered loss of material except SNC 5. However, a smaller loss of material was observed in SNC 2.5 as compared to nSiR and SMC 15. FTIR results were also in agreement with the SEM and hydrophobicity investigations. Better intactness of methyl hydrophobic groups was seen in nanocomposites, but SNC 5 showed greater stability than SNC 2.5. It is quite clear from the investigations that 5 wt%% loading of silica is optimal; it increased the overall stability of the composite and expressed greater resistance against the applied conditions.

## Figures and Tables

**Table 1:** Filler content in prepared composites.

Sample Name	Filler Concentration (wt%)	Sample Code
Neat SiR	0	nSiR
SiR Microcomposites	15	SMC 15
SiR Nanocomposites	2.5	SNC 2.5
SiR Nanocomposites	5	SNC 5

**Table 2:** Prominent groups and relevant absorption bands.

Functional Group	Wave Number (cm^−1^)
Carbon–hydrogen symmetric stretching of CH_3_	~2963–2950
Silicone–CH_3_ bending (symmetric)	~1285–1260
Stretching of Si–O–C	~1115–1060
Stretching of Si–O–Si (asymmetric)	~1140–1010
Silicone-oxygen stretching of O–Si(CH_3_)_3_	~875–860
Silicone-oxygen stretching of O–Si(CH_3_)_2_–O	~850–795
Silicone-carbon stretching of Si–(CH_3_)_3_	~710

**Table 3:** Peak absorption of major groups in nSiR from virgin to 1000 h.

Functional Groups	Wave Number (cm^−1^)	Absorbance					
		Virgin	200 h	400 h	600 h	800 h	1000 h
Carbon–hydrogen symmetric stretching of CH_3_	~2963–2950	0.07	0.01	0.06	0.06	0.07	0.07
Silicone–CH_3_ bending (symmetric)	~1285–1260	0.3	0.08	0.3	0.3	0.3	0.4
Stretching of Si-O-C	~1115–1060	0.4	0.04	0.4	0.5	0.5	0.5
Stretching of Si-O-Si (asymmetric)	~1140–1010	0.5	0.05	0.7	0.9	0.9	0.9
Silicone–oxygen stretching of O–Si(CH_3_)_3_	~875–860	0.1	0.02	0.2	0.2	0.2	0.2
Silicone-oxygen stretching of O–Si(CH_3_)_2_–O	~850–795	0.7	0.06	0.8	1.0	0.9	0.9
Silicone–carbon stretching of Si–(CH_3_)_3_	~710	0.2	0.04	0.2	0.2	0.18	0.2

**Table 4:** Peak absorption of major groups in SMC 15 from virgin to 1000 h.

Functional Groups	Wave Number (cm^−1^)	Absorbance					
		Virgin	200 h	400 h	600 h	800 h	1000 h
Carbon–hydrogen symmetric stretching of CH_3_	~2963–2950	0.07	0.01	0.05	0.09	0.09	0.08
Silicone–CH_3_ bending (symmetric)	~1285–1260	0.3	0.06	0.3	0.4	0.4	0.4
Stretching of Si-O-C	~1115–1060	0.4	0.03	0.4	0.5	0.5	0.5
Stretching of Si–O–Si (asymmetric)	~1140–1010	0.6	0.05	0.6	0.9	0.8	0.9
Silicone-oxygen stretching of O–Si(CH_3_)_3_	~875–860	0.2	0.02	0.2	0.2	0.2	0.2
Silicone–oxygen stretching of O–Si(CH_3_)_2_–O	~850–795	0.7	0.04	0.7	1.0	0.9	1.0
Silicone–carbon stretching of Si–(CH_3_)_3_	~710	0.2	0.02	0.2	0.2	0.2	0.2

**Table 5:** Peak absorption of major groups in SNC 2.5 from virgin to 1000 h.

Functional Groups	Wave Number (cm^−1^)	Absorbance					
		Virgin	200 h	400 h	600 h	800 h	1000 h
Carbon-hydrogen symmetric stretching of CH_3_	~2963–2950	0.07	0.01	0.06	0.08	0.07	0.08
Silicone–CH_3_ bending (symmetric)	~1285–1260	0.3	0.1	0.3	0.4	0.4	0.4
Stretching of Si–O–C	~1115–1060	0.4	0.05	0.4	0.5	0.5	0.5
Stretching of Si-O-Si (asymmetric)	~1140–1010	0.6	0.06	0.7	0.9	0.8	0.9
Silicone–oxygen stretching of O–Si(CH_3_)_3_	~875–860	0.1	0.03	0.2	0.2	0.2	0.2
Silicone–oxygen stretching of O–Si(CH_3_)_2_–O	~850–795	0.8	0.06	0.8	1.0	0.9	0.9
Silicone–carbon stretching of Si– (CH_3_)_3_	~710	0.2	0.06	0.2	0.1	0.2	0.2

**Table 6:** Peak absorption of major groups in SNC 5 from virgin to 1000 h.

Functional Groups	Wave Number (cm^−1^)	Absorbance					
		Virgin	200 h	400 h	600 h	800 h	1000 h
Carbon–hydrogen symmetric stretching of CH_3_	~2963–2950	0.07	0.02	0.06	0.09	0.09	0.09
Silicone–CH_3_ bending (symmetric)	~1285–1260	0.3	0.1	0.3	0.4	0.4	0.4
Stretching of Si–O–C	~1115–1060	0.4	0.06	0.4	0.3	0.3	0.3
Stretching of Si–O–Si (asymmetric)	~1140–1010	0.6	0.09	0.7	0.7	0.7	0.7
Silicone–oxygen stretching of O–Si(CH_3_)_3_	~875–860	0.1	0.03	0.1	0.2	0.2	0.2
Silicone–oxygen stretching of O–Si(CH_3_)_2_–O	~850–795	0.7	0.08	0.7	0.9	0.9	0.9
Silicone–carbon stretching of Si– (CH_3_)_3_	~710	0.2	0.06	0.2	0.2	0.2	0.2

## Data Availability

Data sharing not applicable.
